# AgEvidence: a dataset to explore agro-ecological effects of conservation agriculture

**DOI:** 10.1038/s41597-024-03415-9

**Published:** 2024-06-04

**Authors:** Lesley Atwood, Maria Gannett, Stephen A. Wood

**Affiliations:** 1 The Nature Conservancy, Arlington, VA USA; 2 Cornell Atkinson Center for Sustainability, Ithaca, NY USA; 3 Yale School of the Environment, New Haven, CT USA

**Keywords:** Agriculture, Agroecology

## Abstract

Conservation agriculture (CA) is a set of principles thought to be able to enhance crop productivity while minimizing impacts on the environment. The evidence base for CA can be challenging to synthesize because it encompasses many different practices and social and agroecological outcomes. To facilitate synthesis of CA evidence we have created a dataset organizing 218 response variables from five common categories of CA: cover crops, tillage management, pest management, nutrient management, and crop diversification. These data cover the Midwestern United States (U.S.) from 1980–2020. The dataset is also summarized and visualized on the AgEvidence website, which enables users to interactively explore, filter, and download data. We hope this dataset will help a wide variety of stakeholders, including researchers, policy makers, advocacy groups, and growers access the evidence needed to make informed and impactful decisions about how to produce food with less negative environmental impact.

## Background & Summary

With the looming “triple threat” of climate change, biodiversity loss, and food insecurity^1^, there is an urgent need to increase food production while also reducing negative environmental impact^2–4^. A proposed solution for sustainable intensification of agriculture is conservation agriculture. Conservation agriculture attempts to optimize yield and profit while balancing agricultural, economic, and environmental benefits. This is in contrast to conventional agriculture, which maximizes yields at the cost of environmental resources^5^. The concept of conservation agriculture, as defined in contemporary scientific literature, originated during the dust bowl, experienced across much of North America in the 1930s^6^. In response to the intense wind erosion experienced at this time, there was an effort to reduce erosion through practices such as contour tillage and terracing. In the 1950s, with the introduction of new tillage equipment such as the sweep, the focus shifted to conservation tillage. Conservation tillage was expanded to become conservation agriculture at the Latin-American Network for Conservation Tillage (V RELACO) in 1997. In 1998 the Food and Agriculture Organization of the United Nations (FAO) officially defined conservation agriculture as three primary principles: reduced disturbance, continuous soil cover, and crop rotation^7^. These three principles have been expanded to incorporate integrated soil fertility management^8^ and integrated pest management^5^. Today the term encompasses a wide range of agricultural practices that can be used when appropriate, with the goal of reducing negative environmental impact^5,9^ (Table 1).

**Table 1:** Agricultural practices that can be considered conservation agriculture*.

Category of practice	Conservation agriculture practice
Core conservation ag practices	Increased soil cover
	Minimized soil disturbance
	Crop rotations
Promoting soil cover	Legume fallow
	Agroforestry techniques
Soil improvements	Composting
	Manure
	Organic amendments
Reduced pesticide and fertilizer use	Integrated pest management
	Precision fertilizer applications
	Precision herbicide applications
	Precision insecticide and fungicide applications

*Adapted from Dumanski *et al*.^5^.

Conservation tillage and conservation agriculture have been a focus of agricultural research since the 1930s in the United States (U.S.), but as environmental pressure mounts, this subject has received increasing attention and adoption around the world^10^. Research has helped to improve conservation agricultural practices and to understand their impact on a wide range of agro-environmental variables. With so much information being generated, it is increasingly important to summarize these data to be able to make informed policy and field management decisions that can buffer climate variability and improve ecosystem functioning. Both narrative and meta-analytical reviews are important tools to help understand how conservation practices impact crop production and environmental functioning^11^.

Review papers often focus on a specific management practice and a specific response or set of response variables. A review paper may ask how reduced tillage practices affect greenhouse gas production^12,13^, or how cover cropping affects water quality leaving a field^14^. These reviews offer valuable insights into treatment effects, locations, soil types, and other modulating variables^15,16^. However, they often maintain a narrow focus. It is also important to gather data across conservation practices and across environmental response variables to be able to ask broad-scale questions. These big-picture questions may explore the relationship between different conservation practices, such as: Does tillage or cover cropping have a larger impact on soil organic matter content? Or, these questions may address the field of conservation agriculture, such as: Where are there gaps in the way we understand how conservation agriculture affects agro-ecosystem functioning? These broad-scale questions can quickly become cumbersome to address with a review or meta-analysis due to their large size, although it is possible. The answers to questions such as these would be useful to a wide audience of agricultural professionals, and so to help to begin answering these questions, we created a dataset and web tool to consolidate and organize data on conservation agriculture through AgEvidence^17^.

AgEvidence^17^ is a systematic literature dataset of the multiple benefits and trade-offs associated with multiple conservation agricultural practices in the Midwest U.S. The purpose of this comprehensive assessment is to provide organizations, policy makers, scientists, farmers, and other interested parties with all the available evidence related to the diversity and magnitude of agro-environmental impacts that occur following the implementation of conservation practices. To facilitate exploration and use of these data, we have made our database freely available online at the Knowledge Network for Biocomplexity (KNB) repository and developed an interactive web tool that generates visualizations of these data based on user interests. A similar review that explores the effects of conservation agriculture in Kenya was also conducted but is not described further here.

The overarching question for this research was, what are the agro-environmental benefits and trade-offs associated with the most common conservation practices recommended for large-scale row cropping systems? The conservation practices we focused on included winter cover crops, conservation tillage, strategic nutrient management, early season pest management, and crop rotations in corn and soybean systems. Data were extracted from peer-reviewed literature published between 1980 and 2020 with experimental field sites located in at least one of the following states: Illinois, Indiana, Iowa, Kansas, Michigan, Minnesota, Missouri, North Dakota, Nebraska, Ohio, South Dakota, and Wisconsin. Our dataset includes all response variables reported in each paper where there are isolated comparisons of a conservation practice. With this approach, we were able to capture the state-of-knowledge for a wide diversity of response variables and identify where we lack knowledge.

## Methods

### Management practices

AgEvidence synthesizes the agro-environmental effects of some of today’s most widely used and recommended conservation agriculture practices for row crop agriculture. Currently, management practices include the adoption of winter cover crops, crop rotations, strategic use of fertilizer, integrated early season pest management strategies, and reductions in tillage. For each practice, detailed descriptions and criteria for inclusion in the dataset are provided in Table 2. AgEvidence is a living dataset, meaning that we are continually expanding its scope by adding new conservation practices, increasing the number of years included, and making corrections where necessary. At the time of this publication, the crop rotation set of literature is still in progress. Papers currently included only span the years 2010–2020, data are actively being extracted, and crop rotational data is not yet incorporated into the web tool. In the future, these tasks will be completed, and other improvements will be underway.

**Table 2:** Conservation practice description and inclusion criteria.

Conservation practice	Description & publication inclusion criteria
Winter cover crop	Fall-planted crops that overwinter in the field. These crops are not grown for profit, but for environmental benefits. Cover crop termination occurs in the spring so as not to interfere/compete with cash crop productivity. For each paper, a control treatment of winter fallow (no winter cover crop) must be included. Differences in preceding crops, nutrient additions, and all other management practices among treatments would preclude a paper from the synthesis. Papers that only report treatment differences in cover crop biomass and no other response variables were also excluded.
Reduced tillage	Minimization of soil disturbance and retention of crop residue on soil surface. All tillage type comparisons were included as long as all other management practices were kept constant (e.g. preceding crops and nutrient additions). Tillage practices were later categorized into one of four groups (conventional tillage, conservation tillage, zonal tillage, and no-tillage) based on Reicoisky^31^.
Early season pest management	Insecticides, nematicides, and/or fungicides applied to cropping systems when the cash crop is still in an early vegetative stage (seed-V6). Each paper must include comparisons to a no pesticide control treatment. Pesticides can be applied directly to seeds, soils, or leaves but pesticide application must occur before or while the crop is still in an early season vegetative growth state. Differences in cash crops, nutrient additions, and all other management practices among treatments would preclude a paper. Papers examining invertebrate sensitivity to specific pesticides and pesticide combinations were excluded.
Strategic nutrient management	Fertilizers strategically placed, either spatially or temporally, to optimize crop nutrient uptake while minimizing nutrient loss. Spatially-specific applications could include banding or variable rate application compared with uniform surface broadcasting. Temporally-specific applications could include application during early vegetative growth compared with preplant application or Spring application compared with fall application or split applications compared with single applications. Papers exploring the effect of varying rates or compositions of fertilizer were excluded. Differences in any other management practice among treatments would preclude a paper.
Crop rotation	New crop species planted in a successional pattern on the same field. Each unique crop species added to the sequence (before it begins repeating) is an increased level of crop rotational diversity. For example: corn-soybean-sorghum-soybean = 3 species rotation; alfalfa-alfalfa-corn-wheat = 3 species rotation; corn-soybean-wheat = 3 species rotation. Unplanted crops in the rotation (ex. fallow, grazed field) were not included.

### Literature search

We compiled relevant peer-reviewed research by searching the Web of Science (WoS) database (www.webofknowledge.com). For each search, identical key words were used to describe the geographic locations, cropping system, and years papers were published (Table 3). Unique keywords were used to describe each conservation agriculture management practice (Table 4). Each search for a conservation practice was conducted separately, so the same paper may be included multiple times if the experimental design included several conservation practices. In total, 4,308 papers passed the initial screening for potential inclusion in the dataset.

**Table 3:** Keywords used to search for publications related to each conservation agriculture practice in the Web of Science.

Search	Publication year	Topic 1 (Geographic Location)	Topic 2 (Cropping System)
All	1980–2020	Illinois OR Indiana OR Iowa OR Kansas OR Michigan OR Minnesota OR Missouri OR “North Dakota” OR “South Dakota” OR Nebraska OR Ohio OR Wisconsin OR ((Midwest* AND U.S.) OR (Midwest* AND US) OR (Midwest* AND “United States”))	corn OR maize OR soybean OR “Zea mays” OR “Glycine max” OR agricultur* OR agro-ecosystem* OR agroecosystem* OR crop OR “field crop*“ OR “cropping system” OR farm* OR “conservation agricult*“

These keywords, describing publish date range, geographic location, and cropping system, were used for all four searches. The symbol “*” represents any letter.

**Table 4:** Keywords used to search for publications related to each conservation agriculture practice in the Web of Science.

Search	Topic 3 (conservation practice)	Papers included in synthesis (#)	Total data comparisons (#)
Winter cover crop	“cover crop*“ OR cover-crop* OR covercrop*	85	4,255
Reduced tillage	conservation till* OR “conventional till*“ OR “no-till*“ OR “no till*“ OR “zero till*” OR “zero-till*” OR “reduced till*“ OR “minimum till*“	210	16,714
Early season pest management	pesticide seed treatment* OR “seed treatment*“ OR “systemic insect*“ OR neonic* OR pyrethr* OR (foliar AND insecticide*)	34	1,539
Strategic nutrient management	(precision AND (fert* OR agr* OR nitrogen OR phosphorous)) OR “variable rate” OR “band* fert*“ OR 4 R OR ((enhance* OR efficien*) AND (nitrogen OR phosphorous))	35	1,877
Crop rotation	“crop rotation*“ OR cropdiversi*	60 (2010–2020 still in process)	7,249

These unique keywords identified five conservation practices: winter cover crops, reduced tillage, early season pest management, nutrient management, and crop rotations. The number of papers and the total number of comparisons included in the AgEvidence dataset are also reported. The symbol “*” represents any letter.

All titles and abstracts returned from the initial screening were screened by person. If either the title or abstract provided any reason for exclusion, then it was removed from further screening. If the title and abstract lacked reason for immediate exclusion, the full publication was screened to determine if the paper would be included. The criteria for inclusion in AgEvidence are as follows:

- Peer-reviewed publication (excludes conference proceedings) published between 1980 and December 2020.
- Field study conducted in the Midwest U.S. (excludes laboratory experiments, agricultural models, review papers, qualitative studies, and methodological papers).
- Infield response variables only. Measurements collected beyond the field such as effluence from fields were excluded.
- Cash crops were either corn (including sweet corn), soybean, or both (excludes vegetable, forage, tree, and all other commodity crops). For crop rotation studies, corn or soybean must be included in at least one rotation.
- Experimental treatments must include either winter cover crops, reduced tillage, early season pest management, nutrient management, or crop rotations as described in Table 2.
- Publications were excluded if the treatment of interest was not isolated within the experimental design as these studies can suggest that a management practice could alter the measured outcomes, but they cannot provide direct evidence of a causal relationship between the practice and the observed effect (e.g. when corn-soybean crop rotations are compared to corn-soybean-wheat-winter rye rotations the causal effects of winter rye cannot be disentangled from the effects of wheat, so was excluded from the cover crop dataset, although included in the crop rotation dataset).
- Only quantitative results were extracted. Surveys and qualitative assessments of a treatment were excluded.

**Fig. 1:**
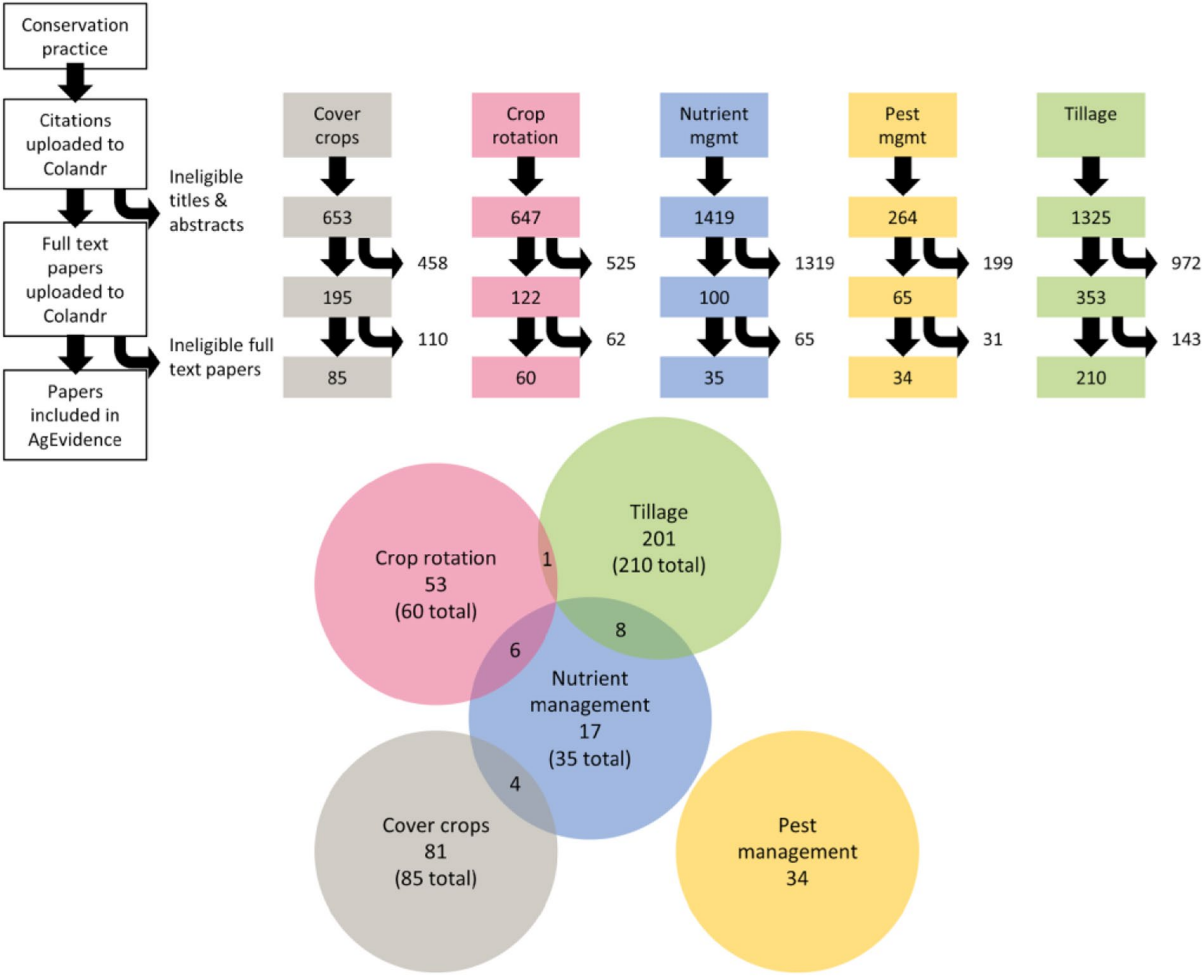
Flow identification and selection of papers for inclusion in the AgEvidence dataset. Each column represents a Web of Science literature search for a different conservation practice studied in the United States (U.S.) Midwest from 1980–2020. The five conservation agricultural practices included are: cover cropping in grey, crop rotations in pink, strategic nutrient management (mgmt.) in blue, pest management in yellow, and reduced tillage in green. The first number in a column shows the number of titles screened after duplication removal in Colandr. The middle number shows the number of number of studies that passed the title and abstract screening. The final number is the number of studies included in the AgEvidence dataset. Numbers to the right show papers screened at each step. Since each literature search was completed separately, some studies that investigate multiple conservation practices are duplicated in the final AgEvidence dataset. The number of duplicate studies shared between conservation practices versus the number of unique studies are represented in the Venn Diagram (circles and overlap are not to scale).

A total of 424 papers passed both the initial title/abstract screening and the full-text screening, 19 of which were included in multiple datasets (Fig. 1). The majority of papers focused on tillage.

### Literature management

For each conservation practice, we downloaded the associated.bib file provided through Web of Science. Then using Colandr^18^ https://www.colandrapp.com/signin, an open-source web platform developed by Conservation International, DataKind, & the Science for Nature and People Partnership (SNAPP), we created a ‘review’ for each conservation practice and uploaded the corresponding.bib file. Colandr is a program designed to assist with literature reviews by providing an organizational framework and implementing machine learning to accelerate the literature screening process. It performs the first screen of uploaded.bib files based on inclusion criteria and by removing duplicates. Title/abstract screening and full text screening were completed after this initial program screen.

Summary statistics were collected primarily from tables and figures. Data from tables were copied directly, and data from figures was captured using Web Plot Digitizer^19^ https://apps.automeris.io/wpd/. All summary response data comparing two different levels of a conservation practice were extracted. If several levels of a conservation practice were included (e.g. no fertilizer, low fertilizer, and high fertilizer use) then each combination was included (e.g. no fertilizer to low fertilizer, low fertilizer to high fertilizer, and no fertilizer to high fertilizer). Comparisons including interaction treatments were extracted if reported in the paper, as well as comparisons averaged across interactions. In total, 24,385 comparisons were collected with a mean of 57.5 comparisons per study. These comparisons are independent within a conservation practice but may be repeated across practices (if data on interactions was included). Response variable data collected was organized into 7 broad categories: climate mitigation, crop yields, economics, other soil properties, pests, soil nutrients, and water quality. These categories were further subdivided twice more into finer and finer levels of detail, resulting in a maximum of 191 categories (Table 5). There is a relatively even spread of data across the 7 response variable categories for cover crops, crop rotation, and tillage. The crop rotation dataset is currently the only dataset including economic response variables, although that is an improvement planned for the future. The crop rotation dataset also does not include many measures of water quality. Nutrient management papers primarily reported crop yield and soil nutrient data and pest management papers primarily reported pest and crop yield data.

**Table 5:** Categories of response variables of data included in the AgEvidence dataset.

Response variable categories		
Level one	Level two	Level three
Climate Mitigation	Carbon Emissions (3), Carbon Storage (3), Nitrogen Emissions (4)	10
Crop Yields	Cover Crop (4), Crop Damage (4), Crop Growth (17), Grain Quality (13), Grain Yield (6), Pesticide Uptake (1), Plant Nutrient Content (11), Stand Count (5), Water Use (3), Yields (3)	67
Economics	Costs (1), Income (2)	3
Other Soil Properties	Abiotic Factors (4), Biotic Factors (12), Chemical Properties (5), Physical Properties (6), Soil Structure (7)	34
Pests	Invertebrate Pests (10), Nematodes (9), Non-Predators & Pests (3), Pathogens (7), Pest Natural Enemies (3), Weeds (18)	50
Soil Nutrients	Ammonium (3), Micronutrients (3), Nitrate (4), Nitrogen (5), Phosphorus & Potassium (2)	17
Water Quality	Agrochemical Runoff (1), Flow Quantity (2), Nutrient Runoff (6), Sediment Runoff (1)	10
Total number of categories at each level		
7	35	191

Data are from studies exploring the effect of five conservation agricultural practices in the United States (U.S.) Midwest from 1980–2020 (cover crops, crop rotation, strategic nutrient management, pest management, and reduced tillage). All data reported in these studies were organized into one of three group level categories. Response level three categories were nested inside the response level 2 categories, which were nested inside the response level one categories. The number in parentheses are the number of response level three categories nested inside each response level two category.

To help users interpret the dataset, normative interpretations for each group were assigned. This was done because an increase in value of some measurements may be undesirable, whereas an increase for others may be desirable. Positive normative values were assigned if the response variable was beneficial to society (e.g. increased soil aggregate size). Negative normative values were assigned if the response variable was detrimental to society (e.g. increased carbon dioxide emissions). Neutral normative values were assigned to response variables if no normative interpretation exists (e.g. sand content) or its interpretation is dependent on the circumstance (e.g. soil nitrogen could be beneficial in nitrogen limited soils, but detrimental to the environment if leaching occurs). Normative values were first assigned at the finest scale of category (group level three). As the user moves up the nested levels, towards increasingly broad categories, the most common normative value in the lower category was used. For example, “Aggregate Size”, “Aggregate Stability”, “Air-filled Pore Space”, “Compaction”, “Total Pore Space”, and “Water-filled Pore Space” are all nested under the group “Soil Structure”. All these categories have a positive normative value because they bring societal benefit except “Compaction” and “Water-filled Pore Space” which have a negative and neutral normative value, respectively. Despite the difference in the normative value of these two categories, the most often assigned normative value to the components of soil structure are positive, so the normative value of “Soil Structure” is positive.

## Data Records

### Dataset structure & Data extraction

Each management practice dataset has six flat csv files with relatable keys available on Knowledge Network for Biocomplexity^20^ (KNB) https://knb.ecoinformatics.org/view/doi%3A10.5063%2FF16Q1VQP. These datasets have a few unique columns and are otherwise similarly structured. In general, each dataset is saved as six csv files named Reference, ExpD_Location, CashCrop, Treatment, Results, and Metadata. The Reference file contains bibliographic information for each publication. The ExpD_Location file contains experimental design, replications, plot size, experimental year(s), and field site location information. The CashCrop file includes information about the cash crop such as preceding tillage practice, seeding density, cultivar details, and crop genetics. The Treatment file includes a description of each treatment. Columns on this sheet vary by management practice. The Results file includes details about response variables, measured values, statistical findings, and response variable groupings. All response data were converted to percent change with Eq. (1):

1 $$\Delta V=\frac{{T}_{2}-{T}_{1}}{{T}_{1}}\times 100{\rm{ \% }}$$

Where Δ*V* is the percent change in response variable, *T*_2_ is the mean value when the conservation practice is enacted (treatment), and *T*_1_ is the mean value when the conventional practice is in enacted (control). When multiple levels of conservation practice are tested (e.g. one-crop, two-crop, and three-crop rotations) *T*_1_ is always the more conventional practice (e.g. one-crop and two-crop) and *T*_2_ is always the more conservation-oriented practice (e.g. two-crop and three-crop, respectively). When the response variables are already reported as a percent, then the percent change, Δ*V*, is calculated using Eq. (2):

2 $$\Delta V={T}_{2}-{T}_{1}$$

Finally, Metadata for all column names are included as a separate file within each management practice dataset. This information is also provided in the Supplementary Information (Tables S1-S11).

## Technical Validation

The primary objective of our data validation process was to ensure the precision of the data collected from peer-reviewed scientific literature. This was achieved through a series of checks: (1) multiple data collectors to cross-verify information, (2) the identification and verification of outliers, and (3) data validation facilitated by end-user notifications. We should note that we did not evaluate the inherent accuracy of data within the studies themselves, as only peer reviewed studies were included in our dataset. The peer-review process provides a baseline level of data accuracy, however, there can still be inherent biases in published literature^21–24^. Data validation at the level of the publication was beyond the scope of this review.

To ensure that data were accurately extracted from research articles 10% of all studies were independently collected by two researchers. Discrepancies between their collected data were identified and discussed. Any differences in data collection practices were resolved to improve the consistency of data collection. This process was done separately for each set of conservation agricultural practice studies.

Following data collection, datasets were analysed to identify potential outliers. These outliers were detected either by isolating data points with unusually high or low values using the AgEvidence web tool, or by identifying data points with z-scores exceeding 3.29 or below −3.29 (function *scale* in base R, version 4.3.0). The z score is calculated using Eq. (3):

3 $${z}_{i}=\frac{{x}_{i}-\underline{X}}{{s}_{x}}$$

where $\mathop{\,\,{X}_{1}}\limits_{\bar{} }$ is the mean of X and *s*_*x*_ is the standard deviation for data with a normal distribution. All data analyses were done using R statistical software version 4.3.0^25^.

These potential outliers were then verified from the data reported in the primary literature. Potential outliers were identified for treatment, control, and response data (percent change). Potential response data outliers were checked by verifying numbers reported for both the treatment and the control. Identifying and verifying potential outliers is efficient in two ways. It isolates the data that are most likely to be misrepresented. These data also have the greatest potential influence on analyses and therefore it is important to ensure their accuracy.

After these validation steps, the data were integrated into the publicly accessible AgEvidence web tool www.agevidence.org. This platform allows users to interact with the data based on their specific interests. As users explore the dataset, sometimes they encounter discrepancies and reach out for clarification. We confirm the accuracy of any data that users may have questions about and resolve any errors.

We also explored the bibliographic information of papers included in AgEvidence as another component of our data validation process^26,27^. Tillage was the most well represented conservation practice in our database. Studies focusing on tillage made up 52% of the publications (210 out of 405 papers). Nutrient management, which only accounted for 8.6% of the publications (35 out of 405 papers) also shared the most papers with other conservation practices (18 shared papers out of 35 papers total) (Fig. 1). Within each conservation practice, results from all response variable categories were measured and reported except nutrient management and pest management. Those two conservation practices focused predominantly on reporting yield data and data directly related to the practice (soil nutrients for nutrient management and pests for pest management). Going forward, it may be useful to explore the effects of early season pest management and strategic nutrient management on climate mitigation, other soil properties, and water quality. The effects of nutrient and pest management on those categories of ecological responses are currently underexplored in the literature included in AgEvidence.

To explore the authorship community, we used a co-occurrence network of the authors included within the AgEvidence dataset (function *graph_from_adjacency_matrix*, in package igraph, version 1.5.1). Research groups can introduce bias into their research outcomes. These biases arise from specific methodologies and protocols frequently used by different research groups^28^, laboratory conditions^29^, composition and size of the research team^30^, and the centrality of collaborations between research groups^28^. The examination of authorship communities remains a relatively new area of study, making it currently difficult to identify potential points of bias from the author network connectivity. However, we hope this type of exploration becomes more common in future analyses. The code for this analysis is publicly available at GitHub https://github.com/mag449/AgEvidenceMethodsCode, enabling future users of AgEvidence to readily investigate the author community relevant to their own research priorities.

**Fig. 2:**
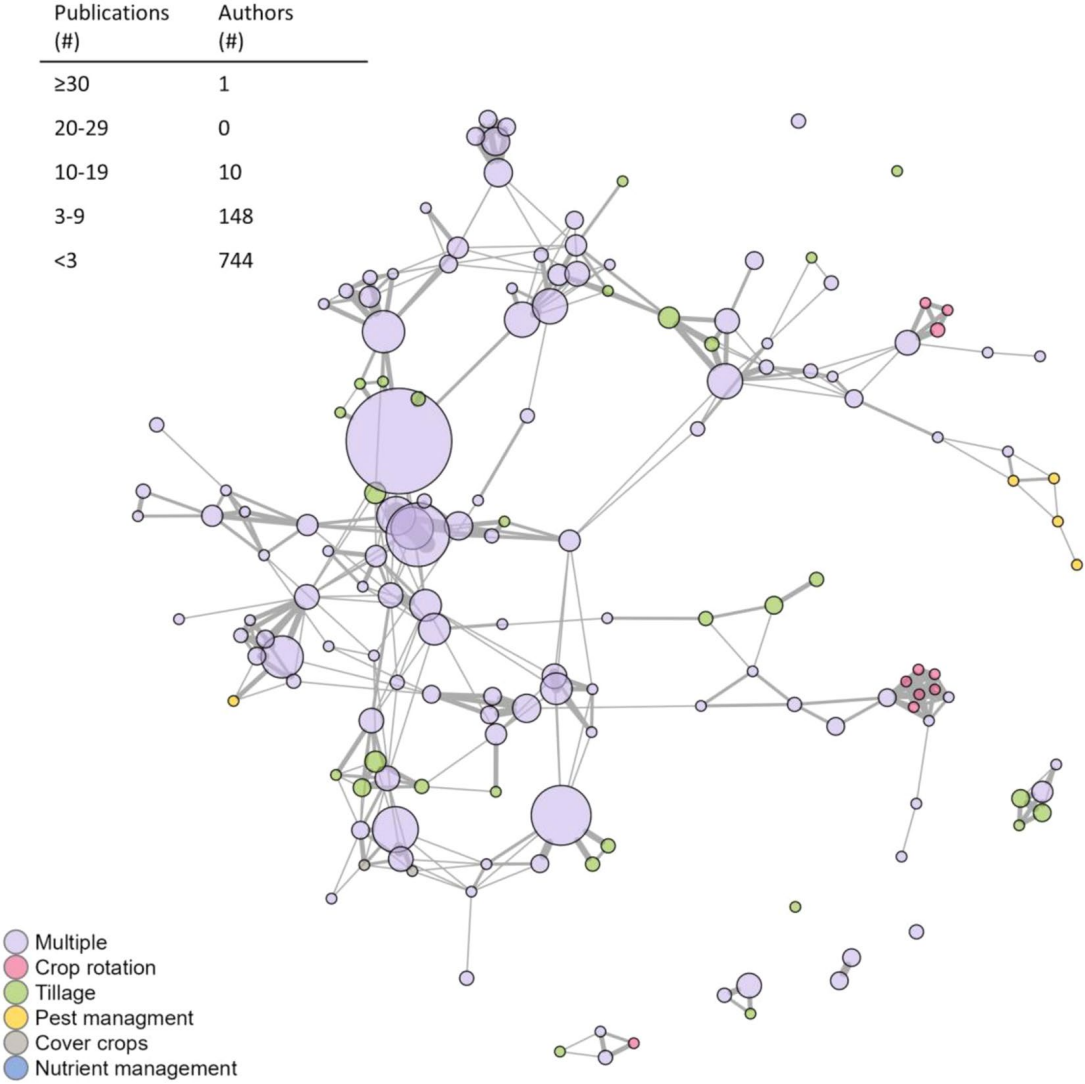
Network analysis of authorship communities included in AgEvidence. Circles (nodes) in the co-occurrence network represent authors who have published more than 2 papers on conservation agriculture in the Midwest United States (U.S.) between 1980 and 2020. This represents 159 out of the 903 authors total. The size of the circle corresponds to the number of papers they have published. Grey lines (edges) connect authors who have been co-authors. The width of the edge represents the number of times those two authors have published together. Colors of circles indicate the type of conservation practice that an author published about where ‘Multiple’ refers to authors who have published about multiple conservation practices. The number of authors who have published different numbers of papers are listed in the table.

Our network analysis includes authors who have published more than two papers in the AgEvidence database. This represents 8.4% of the total authors (159 out of the 1,686) but this cut-off improves the readability of the authorship network while still capturing important authorship relationships (Fig. 2). One author was involved in 7% of all the papers (30 out of 405 papers) in the AgEvidence database; significantly more than any other author (Fig. 2). All other authors were involved in 4% or less of the papers in AgEvidence (18 out of 424 papers or fewer), and the median and mode number of papers an author published was one (0.2% of papers). Even though most papers in our dataset focus on tillage, most authors included in the network published papers on multiple conservation practices. This provides assurance that although tillage may be disproportionately represented, authors are experts in conservation agriculture broadly, not just conservation tillage. There were, however, a few clusters of authors focused around one practice. Most authors were connected to the largest interconnected network, and only four authors (nodes) were completely disconnected from other authors.

### Supplementary information

Supplementary Information

## Data Availability

All code for calculating percent change and all analyses included in this manuscript can be found at GitHub https://github.com/mag449/AgEvidenceMethodsCode.
