# Supplementary material for: AgEvidence: a dataset to explore agro-ecological effects of conservation agriculture
Source: Sci Data. 2024 Jun 4;11:581. doi: 10.1038/s41597-024-03415-9 (PMC11150489; doi:10.1038/s41597-024-03415-9)
Supplement: Supplementary file 1 — Supplementary Information [file 41597_2024_3415_MOESM1_ESM.pdf]

Supplementary Information

Table of Contents

Table S1. Metadata for Reference file. 1
Table S2. Metadata for Experimental Design & Location files. 2
Table S3. Metadata for Cash Crop files. 3
Table S4. Metadata for Treatment files. 4
Table S5. Metadata for Results files. 8
Table S6. Tillage types with ranking number (Results:tillage_1 and tillage_2) and Results:fine_levelgroup options. 12
Table S7. Cover crop options for Results:fine_levelgroup and Results:cc_group1 with descriptions. 13
Table S8. Early Season Pest Management options for Results:fine_levelgroup. 13
Table S9. Nutrient management labels for Results:nutrient_groups, trt1_name, and trt2_name. 14
Table S10. Cover crop labels for Results:cc_group2 which describe the functional diversity of the cover crops included in the treatment. 14
Table S11. Early season pest management labels for Results:pm_group1 which describe the pesticides included in the treatment. 14

Table S1. Metadata for Reference file.

Review	Column Name	Relational Key	Description
ALL	paper_id	ALL	Unique identification number for this manuscript
ALL	doi	ExpD_Location	Digital object identifier (permanent and unique string of characters that links to the publication)
ALL	authors		Authors listed as Smith, A.B, Jones, C.D., & Clarke, E.F.
ALL	authors_abbrev		Last et al. OR Last & Last
ALL	pubyear		Year publication was printed
ALL	journal		Journal name
ALL	volume_issue		Journal volume number, if there is an issue # include it as Volume (Issue) (e.g. 62(1))
ALL	title		Complete title of article (first letter of first word capitalized; all other words lower case except proper nouns)
ALL	pages		Journal page numbers (e.g. 10-15)
ALL	readername		Initials of individual who read and extracted data
ALL	dateread		Date the reader read and extracted these data
ALL	notes		Comments about the article

Table S2. Metadata for Experimental Design & Location files.

Review	Column Name	Relational Key	Description
ALL	doi	Reference	Same as 'Reference:doi'
ALL	paper_id	ALL	Unique identification number for this manuscript. Same as 'Reference:paper_id'.
ALL	loc_multi		Number assigned to each unique experimental location. Start a new row for each unique location (experiments with multiple sites). 'paper_id' remains the same for each site.
ALL	soil_type		Description of soil type provided by authors in manuscript
ALL	city		Name of city or research station associated with research site location
ALL	state		State associated with research site location
ALL	latitude		Latitude associated with research site location
ALL	longitude		Longitude associated with research site location
ALL	year_start		Year first cash crop was planted for the study
ALL	years_num		Total number of years study was conducted (from planting of first cash crop to harvest of final cash crop)
ALL	annual_precip		Average annual precipitation (mm) for study site (record only if reported in manuscript)
ALL	field_history		Any field history reported for the study site (i.e. 30-year history of fallow prior to study, all treatments received manure before the start of the experiment, etc.)
Tillage	years_tillage_implemented		Total number of years tillage was implemented at the site. This number may exceed 'years_num'.
ALL	exp_design		Experimental design description (e.g. latin square, randomized complete block (RCBD), site comparison, paired, before-and-after)
ALL	exp_arrangement		Describe experimental design arrangement (e.g. split plot, split-split-plot etc.)
ALL	reps		# of replications

ALL	plot_width		Plot width for the treatment of interest. If unclear, report smallest plot width. Report all dimensions in meters.
ALL	plot_length		Plot length for the treatment of interest. If unclear, report smallest plot length. Report all dimensions in meters.
ALL	trtmt_levels		Total number of treatments included in study (main level only = 1, split plot = 2, split-split plot = 3, etc.)
ALL	trtmt_main		Description of main plot treatment (e.g. Tillage)
ALL	trtmt_main_levels		Number of levels within main treatment (e.g. moldboard plow, chisel plow, no-tillage = 3 levels)
ALL	trtmt_splitA		Description of split plot treatment (e.g. Cover Crops)
ALL	trtmt_splitA_levels		Number of levels within split treatment (e.g. no cover crop, winter rye, winter rye + hairy vetch = 3 levels)
ALL	trtmt_splitB		Description of split-split plot level treatment (e.g. Fertilizer)
ALL	trtmt_splitB_levels		Number of levels within split-split treatment (e.g. 0 kg/ha, 50 kg/ha, 100 kg/ha = 3 levels)
ALL	trtmt_splitC		Description of split-split-split plot level treatment (e.g. Seed Treatment)
ALL	trtmt_splitC_levels		Number of levels within split-split-split treatment (e.g. untreated, insecticide-fungicide seed treatment = 2 levels)

Table S3. Metadata for Cash Crop files.

Review	Column Name	Relational Key	Description
ALL	paper_id	ALL	Unique identification number for this manuscript. Same as 'Reference:paper_id'.
ALL	duration	Treatment & Results	Start year ('ExpD_Location:year_start') - Length of experiment ('ExpD_Location:years_num')
ALL, except Crop Rotation	cash_tillage		Describe tillage practice that occurred prior to cash crop planting (e.g. chisel, moldboard, no-tillage)

ALL, except Crop Rotation	cash_seeddensity	Cash crop seeding density (# seeds/ha)
ALL	cash_species	Species of cash crop (must be either Zea mays and/or Glycine max)
ALL, except Crop Rotation	cash_cultivar	Specific name(s) of cash crop cultivars planted
ALL, except Tillage and	cash_genetics	Description of cash crop genetics (e.g. glyphosate resistant (GR))
Crop Rotation	cropID	ID number assigned to each crop species, starting at 1 for each new paper. There should be one species per row.
Crop Rotation	crop_species	Name of crop species. There should only be one species per row.
Crop Rotation	tillage	Describe tillage practice that occurred prior to crop planting (e.g. chisel, moldboard, no-tillage), write "variable" if tillage is an interaction.
Crop Rotation	plant_date	Approximate date of planting for the crop species
Crop Rotation	plant_implement	Planting method (i.e., no-till drilled, broadcasted) or tractor type
Crop Rotation	seed_density	Seeding density of the species. Include units for each species.
Crop Rotation	cultivar	Specific name(s) of crop cultivars planted
Crop Rotation	genetics	Description of crop genetics (e.g. glyphosate resistant (GR))
Crop Rotation	fertilizer	Quantity and type of fertilizer applied to the crop species
Crop Rotation	notes	Any additional information regarding the management practices for the crop species

Table S4. Metadata for Treatment files.

Review	Column Name	Relational Key	Description
ALL	paper_id	ALL	Unique identification number for this manuscript.
ALL	duration	CashCrop & Results	Start year ('ExpD_Location:year_start') - Length of experiment ('ExpD_Location:years_num')

ALL	trt_id	Unique identification number for each treatment included in study. Control treatment = 0. Control treatment is the absence of the treatment of interest (i.e. for cover crop review Control = No Cover Crop).
ALL	year	Year(s) in which specific treatment was applied, if all years of the study (0); if deployed only in first year = 1, only in second year = 2, etc.
Tillage	till_type	Describe tillage treatment
Tillage	till_depth_cm	Tillage disturbance soil depth (cm)
Tillage	till_numpasses	Total number of tillage passes prior to planting
Tillage	till_dates	Dates tillage was performed
Tillage	trt_name	Name given to treatment in publication
Tillage, Cover Crop, Nutrient Management	trt_other_description	Description of any other details associated with the treatment (e.g. corn rotation, seed treatment applied, etc.)
Cover Crop	cc_soilprep	Description of soil preparation prior to planting cover crops (often tillage types)
Cover Crop	cc_plantdate	Date or season cover crops were planted
Cover Crop	cc_plantimplement	Cover crop planting method (i.e., no-till drilled, broadcasted)
Cover Crop	cc_fertilizer	Quantity and type of fertilizer applied specifically for cover crops
Cover Crop	cc_max_diversity	Maximum diversity of cover crops used in this paper (i.e., single or mixture)
Cover Crop	cc_mixture	Description of specific cover crop species or mixture of species for this treatment. If no cover crop, use 'no cover crop'.
Cover Crop	cc_cultivar	Cultivar name(s) for cover crop species. Listed in same order as species list in 'cc_mixture'
Cover Crop	cc_seeddensity	Cover crop seeding density (include units). If treatment includes multiple species, list the seeding density for each species in the same order the species are described in 'cc_cultivar'. If the same species is seeded at multiple rates, list all rates.

Cover Crop	termination	Method used to terminate cover crops (e.g. herbicide, tillage, etc.)
Cover Crop	herbicide	If cover crops were terminated with herbicides, list the type(s) of herbicide used.
Cover Crop	herbicide_rate	Rate herbicides were applied to terminate cover crops. If multiple herbicides were used, list the rates in the same order as the list of 'herbicide's.
Cover Crop	herbicide_rateunits	Units associated with 'herbicide_rate'
Cover Crop	termination_timing	Date cover crops were terminated. Along with actual dates, a description of termination time relative to planting of cash crop is helpful.
Nutrient Management	fert_app	Describe fertilizer application method (e.g. broadcast, banded, split application, single, etc.)
Nutrient Management	fert_type	Description of fertilizer type applied
Nutrient Management	fert_rate	Fertilizer application rate. If variable rate, state 'variable rate'.
Nutrient Management	fert_rate_unit	Units associated with 'fert_rate'
Nutrient Management	fert_date	Date of fertilizer application
Nutrient Management	trt_name	Name treatment is given in the publication
Early Season Pest Management	pest_trtmtdescription	Broad description of pesticide treatment used (e.g. untreated, fungicide-insecticide seed treatment, foliar insecticide). If a combination of treatments were used use '+' in-between each treatment description.
Early Season Pest Management	pest_tradename	Insecticide(s) tradename(s). If there are multiple use '+' in-between tradenames. Include 'fungicides' if any fungicides were included in the pesticide treatment.
Early Season Pest Management	pest_fungicide	List of fungicides and application rates included in pesticides.
Early Season Pest Management	pest_insecticides	List of insecticides and application rates included in pesticides.

Early Season Pest Management	pest_application	Detailed description of pesticide application event. This description may include, but is not limited to, application site, pest pressure, or time of season.
Early Season Pest Management	trt_other	Description of any other details associated with the treatment (e.g. seeding rates, co-applied herbicides)
Crop Rotation	treatment	General name of the type of treatment we are interested in (crop rotation, tillage, fertilizer, seed genetics, pesticide use, etc.). These will be the same catagories as those listed in ExpD_Location 'trtmt_levels' but will correspond with the comparisons we're interested in, not the article authors. We may not list all the catagories the authors are intersted in - only those where the crop rotation treatment data is not reported seperately from another interaction. Crop rotation will always be the first treatment listed.
Crop Rotation	int_level	Options are: 'trt' or 'int'. Crop rotation levels are always 'trt' and all additional treatments are labeled as 'int' (i.e. interaction terms)
Crop Rotation	trt_description	General description of the treatment listed in the 'treatment' column. For crop rotation levels (trt) always write out the number of unique crop species included in the rotation such as: one crop, two crops, three crops. Use appropriate descriptors for interaction terms (int) such as: moldboard plow, no till, high fertilizer, low fertilizer. Always start with the most conventional practice (lowest trt_id number) and end with the most improved practice (highest trt_id number).
Crop Rotation	crop_IDs	Leave blank if this row an interaction term (int). If this row is a crop rotation treatment (trt) list the corresponding CropIDs from the Crops tab of the species involved in the crop rotation. Put '-' between

			crop species in a rotation, put ';' between different rotation patterns.
Crop Rotation	trt_details		For crop rotation treatments (trt) list the names of the crop species indicated by the crop_IDs. Seperate species with '-' and rotation patterns with ';' to match the crop_ID column. For interaction terms (int), include any additional relevant details about the treatment.

Table S5. Metadata for Results files.

Review	Column Name	Relational Key	Description
ALL	review		Name of review: Cover crop, Tillage, Early Season Pest Management, Nutrient Management, Crop Rotation
ALL	paper_id	ALL	Unique identification number for this manuscript. Same as 'Reference:paper_id'.
ALL	duration	CashCrop & Treatment	Start year ('ExpD_Location:year_start') - Length of experiment ('ExpD_Location:years_num')
ALL	rv_year		Numeric year associated with results. Instead of reporting year, report the numeric year for each result relative to start year (e.g. if experiment was conducted from 1990-1995 and the result is for 1993, record '4' [count 1990, 1991, 1992, 1993]. If result is an average for multiple years, list all-inclusive year numbers (e.g. avg of years 8 to 10 = 8;9;10). Use ; to separate numeric years.
ALL	loc_multi_results	Expd_Location	Use same number assigned to the experimental site found at 'ExpD_Location:loc_multi'. Report '0' if the results are averaged across all sites.
ALL	group_level1		Group name used to broadly describe response variables: Climate Mitigation, Crop Production, Other Soil Properties, Soil Nutrients, Water Quality

ALL	group_level2	Group name used to describe subgrouping of response variables. To access preexisting list, filter database by group_level1 and view options for group_level2. Additional subgroupings can be added if none of the existing subgroupings accurately describe the response variable.
ALL	group_level3	Group name used to describe sub-subgrouping of response variables. To access preexisting list, filter database by group_level1 and group_level2 and view options for group_level3. Additional subgroupings can be added if none of the existing subgroupings accurately describe the response variable.
ALL	rv	Response variable measured (e.g. yield, SOM, total soil carbon, etc.)
ALL	rv_depth	Soil sampling depth reported in publication for the response variable (often reported in Methods section). If not applicable, leave blank.
ALL	sample_depth	Soil sampling depth groups used to organize response variables. Preset options include 0-30 cm, 0-60 cm, 0-100 cm, 0-150 cm. Choose a soil group that includes rv_depth (i.e., rv_depth = 15-45 cm, sample_depth = 0-60 cm)
ALL	rv_units	Units associated with response variable measured (use ^ before a number to designate it as an exponent). For SOC data, if available, collect values reported on a mass/area/yr basis OR mass/area reported annually. This allows for standardization of these data to Mg C/ha/yr.
ALL	stat_test	Statistical test performed (e.g. ANOVA, Tukeys, LSD)
ALL	stat_type	Statistical value type reported (e.g. mean, sem)

ALL	trt1	Treatment	Control treatment. Numbers assigned here correspond to descriptions provided in 'Treatment:Trt_id'. Control treatments are the more conventional practice of the two practices being compared (e.g. Tillage = Moldboard plow, Cover Crop = No cover crop, Nutrient Management = Broadcast, Single Application, or Fall).
ALL	trt1_int	Treatment	Interaction treatment. Numbers assigned here correspond to descriptions provided in 'Treatment:Trt_id'. If there is no interaction, leave blank. This same number should be used for 'Results:trt2_int'.
ALL	trt1_int2	Treatment	Secondary interaction treatment. Numbers assigned here correspond to descriptions provided in 'Treatment:Trt_id'. If there is no interaction, leave blank. This same number should be used for 'Results:trt2_int2'
ALL	trt1_value		Value reported for 'trt1' and, if applicable, interacting treatments
ALL	trt2	Treatment	Treatment treatment. Numbers assigned here correspond to descriptions provided in 'Treatment:Trt_id'. Treatment treatments are the more conservational practice of the two practices being compared.
ALL	trt2_int	Treatment	Interaction treatment. Numbers assigned here correspond to descriptions provided in 'Treatment:Trt_id'. If there is no interaction, leave blank. This same number should be used for 'Results:trt1_int'.
ALL	trt2_int2	Treatment	Secondary interaction treatment. Numbers assigned here correspond to descriptions provided in 'Treatment:Trt_id'. If there is no interaction, leave blank. This same number should be used for 'Results:trt1_int2'.
ALL	trt2_value		Value reported for 'trt2' and, if applicable, interacting treatments

ALL	significance	For statistical tests, alpha value reported
ALL	normative_effect	Normative interpretation of conservation practice effect where -1 = bad outcome, 0 = no effect, 1 = good outcome.
ALL	finelevel_group	Abbreviated description of comparison between control and treatment. See below for list of shorthand for Tillage (Supplementary Table 6), Cover Crop (Supplementary Table 7), and Early Season Pest Management (Supplementary Table 8).
ALL	trt1_name	Abridged qualitative description of trt1. For Nutrient Review, see Supplementary Table 9.
ALL	trt1_details	Unabridged description of trt1
ALL	trt2_name	Abridged qualitative description of trt2. For Nutrient Review, see Supplementary Table 9.
ALL	trt2_details	Unabridged description of trt2
Tillage	tillage_1	Numeric value assigned to each tillage practice based on Reicoisky, 2015. See Supplementary Table 6 below.
Tillage	tillage_2	Numeric value assigned to each tillage practice based on Reicoisky, 2015. See Supplementary Table 6.
Tillage, Crop Rotation	trt_compare	Description of the two tillage treatments or crop rotation levels being compared
Cover Crop	cc_group1	Description of treatment comparisons for the row. See Supplementary Table 7 below.
Cover Crop	cc_group2	Description of 'trt2' plant functional trait (e.g. Non-legume, Legume, Non-legume + Legume Mixture, Legume Mixture). See Supplementary Table 10 below.
Nutrient Management	nutrient_groups	Do the treatments being compared vary in 'Application Timing' OR 'Fertilizer Placement'? See Supplementary Table 9 below.
Early Season Pest Management	pm_group1	Broad classification of pesticide(s) used. See Supplementary Table 11 below.
Early Season Pest Management	pm_group2	Pesticide application site (e.g. Seed, Soil, Foliar, or combinations of these)

Crop Rotation	years_compare	Description of the two levels of crop rotation measured in years.
Crop Rotation	trt2_group2	Description of 'trt2' plant functional traits (Non-legume or Legume), crop species separated by '-'.
Crop Rotation	lifecycle_group2	Description of 'trt2' plant lifecycle (annual or perennial), crop species separated by '-'.
Crop Rotation	winter_cover	Y if one of the crops in rotation in group2 is a winter annual or perennial (mostly winter wheat or alfalfa), N if there is no winter cover included.
Crop Rotation	Interseeded	Y if one of the crops in rotation in group2 was interseeded into another crop in rotation, N if not.

For some columns in the Results worksheet there are pre-set label options. These columns include: fine_levelgroup, tillage_1, tillage_2, cc_group1, cc_group2, nutrient_groups, trt1_name, trt2_name, pm_group1, and pm_group2. The following tables provide lists of these label options.

Table S6. Tillage types with ranking number (Results:tillage_1 and tillage_2) and Results:fine_levelgroup options.

Rank tillage_1, tillage_2	Tillage type	'Results:fine_levelgroup'	Simplified groupings
0	Conventional tillage – Authors do not explicitly state the type of conventional tillage used	Conventional	Conventional
1	Moldboard plow	MP	Conventional
2	Disc plow	Disc	Conventional
3	Deep ripper	Deep	Conventional
4	Subsoil-HD	Subsoil	Conventional
5	Rotary tillage	Rotary	Conventional
6	Chisel plow	CP	Conservation
6.5	Conservation tillage – Authors do not explicitly state the type of conservation tillage used	Conservation	Conservation
7	Field cultivator	Cultivator	Conservation
7.5	Deep zonal tillage	Deepzone	Zonal
8	Ridge tillage	RT	Zonal
9	Subsoil-LD	subsoil_low	Conservation
10	Vertical tillage	Vertical	Conservation
11	Reduced tillage	Reduced	Conservation
12	Mulch tillage	Mulch	Conservation
13	Stubble mulch	Stubble	Conservation
14	Strip tillage	ST	Zonal

15	Slot tillage	Slot	Zonal
16	No tillage – LD & HD	NT	No-tillage

Table S7. Cover crop options for Results:fine_levelgroup and Results:cc_group1 with descriptions.

‘Results:fine_levelgroup’	‘Results:cc_group1’	Description
Mono	Single species	Single cover crop species compared with no cover crop
mono_mono	Mixture comparisons	Single cover crop species compared with single cover crop species
mono_mix_2	Mixture comparisons	Single cover crop species compared with mixture of two cover crop species
mono_mix_3	Mixture comparisons	Single cover crop species compared with mixture of three cover crop species
mix_2	Two species	Mixture of two cover crop species compared with no cover crop
mix_2_mix_2	Mixture comparisons	Mixture of two cover crop species compared with mixture of two cover crop species
mix_2_mix_3	Mixture comparisons	Mixture of two cover crop species compared with mixture of three cover crop species
mix_3	Three or more species	Mixture of three or more cover crop species compared with no cover crop

Table S8. Early Season Pest Management options for Results:fine_levelgroup.

For each label, “I” indicates insecticides, “F” indicates fungicides, and “N” indicates nematicides as part of the pesticide treatment.

Results:fine_levelgroup	Description
untreated	No pesticides applied (Control treatment)
bandF or bandI	Banded in soil near cash crop
broadcastF or broadcastI	Broadcasted uniformly in field
foliarF, foliarI, or foliarIF	Foliar application when plant has emerged
furrowF, furrowI, or furrowIF	Banded in soil in crop interrow/furrow
seedF, seedI, seedIF, seedIFN,	Seeds pre-coated prior to planting
seedfoliarI	Combination of seeds pre-coated prior to planting and foliar application when plant has emerged
soilF, soilI, soilIF	Drenching soil in and around cash crop

Table S9. Nutrient management labels for Results:nutrient_groups, trt1_name, and trt2_name.

For each nutrient_groups listed, the possible trt1_name and trt2_name options are preceded by the same numeral.

Column Name	Label Options	Description
nutrient_groups	1. Application Timing<br>2. Fertilizer Placement	1. Comparison of fertilizer application timing<br>2. Comparison of fertilizer placement
trt1_name	1a. Fall<br>1b. Preplant<br>1c. Single Application<br>2a. Broadcast	1a. Fertilizer applied in fall preceding planting<br>1b. Fertilizer applied in spring preceding planting<br>1c. Fertilizer applied in one application<br>2a. Fertilizer broadcast throughout field
trt2_name	1d. Spring<br>1e. Early Season<br>1f. Split Application<br>2b. Banded<br>2c. Variable Rate Application	1d. Fertilizer applied in spring<br>1e. Fertilizer applied in spring after planting<br>1f. Fertilizer applied in two or more applications<br>2b. Fertilizer banded either in rows or interrows<br>2c. Fertilizer rate variable throughout field

Table S10. Cover crop labels for Results:cc_group2 which describe the functional diversity of the cover crops included in the treatment.

Results:cc_group2	Description
Legume	All cover crop species included are leguminous. Only use when cc_group1 = Single species and Mixture comparisons.
Non-Legume	All cover crop species included are non-leguminous. Only use when cc_group1 = Single species and Mixture comparisons.
Non-Legume Mixture	All cover crop species mixtures included are non-leguminous mixtures. Only use when cc_group1 = Two species, Three or more species, and Mixture comparisons.
Non-Legume + Legume Mixture	All cover crop species mixtures included are non-leguminous + leguminous mixtures. Only use when cc_group1 = Two species, Three or more species, and Mixture comparisons.
Rotation of Cover Crops	When results spanning multiple years are reported and the cash crop and/or cover crop species varies by year.

Table S11. Early season pest management labels for Results:pm_group1 which describe the pesticides included in the treatment.

Results:pm_group1	Description of pesticide treatment
Fungicide	Only fungicides (single type or mixture of types)

Neonicotinoid (Insecticide)	Only insecticides of class Neonicotinoid
Neonicotinoid & Fungicide	Insecticides of class Neonicotinoid combined with fungicide(s)
Neonicotinoid & Pyrethroid (Insecticide)	Insecticides of class Neonicotinoid combined with Insecticides of class Pyrethroid
Organophosphate (Insecticide)	Insecticides of class Organophosphate
Organophosphate & Pyrethroid (Insecticide)	Insecticides of class Organophosphate combined with Insecticides of class Pyrethroid
Organophosphate, Pyrethroid, & Fungicide	Insecticides of class Organophosphate combined with Insecticides of class Pyrethroid and fungicide(s)
Other Insecticides	All insecticides other than those classified as Neonicotinoids, Organophosphates, or Pyrethroids
Pyrethroid (Insecticides)	Insecticides of class Pyrethroid
